# A Comprehensive Landscape of Imaging Feature-Associated RNA Expression Profiles in Human Breast Tissue

**DOI:** 10.3390/s23031432

**Published:** 2023-01-28

**Authors:** Tian Mou, Jianwen Liang, Trung Nghia Vu, Mu Tian, Yi Gao

**Affiliations:** 1 School of Biomedical Engineering, Medical School, Shenzhen University, Shenzhen 518000, China; 2 Department of Medical Epidemiology and Biostatistics, Karolinska Institutet, SE 17177 Stockholm, Sweden

**Keywords:** nondiseased breast tissue, imaging genomics, pathological image features, bioinformatics, gene expression

## Abstract

The expression abundance of transcripts in nondiseased breast tissue varies among individuals. The association study of genotypes and imaging phenotypes may help us to understand this individual variation. Since existing reports mainly focus on tumors or lesion areas, the heterogeneity of pathological image features and their correlations with RNA expression profiles for nondiseased tissue are not clear. The aim of this study is to discover the association between the nucleus features and the transcriptome-wide RNAs. We analyzed both microscopic histology images and RNA-sequencing data of 456 breast tissues from the Genotype-Tissue Expression (GTEx) project and constructed an automatic computational framework. We classified all samples into four clusters based on their nucleus morphological features and discovered feature-specific gene sets. The biological pathway analysis was performed on each gene set. The proposed framework evaluates the morphological characteristics of the cell nucleus quantitatively and identifies the associated genes. We found image features that capture population variation in breast tissue associated with RNA expressions, suggesting that the variation in expression pattern affects population variation in the morphological traits of breast tissue. This study provides a comprehensive transcriptome-wide view of imaging-feature-specific RNA expression for healthy breast tissue. Such a framework could also be used for understanding the connection between RNA expression and morphology in other tissues and organs. Pathway analysis indicated that the gene sets we identified were involved in specific biological processes, such as immune processes.

## 1. Introduction

The nucleus is the regulatory center of cell inheritance and metabolism, and it contains almost all cellular genomes. Gene expression is the most basic level in genetics, at which a genotype generates a phenotype. The morphological interpretation of histological images of tissue samples is essential for characterizing complex histology imaging phenotypes. Previous studies have focused on the association between the nuclear phenotype and gene expression among serious diseases, e.g., breast cancer [1,2,3,4,5]. However, the gene expression pattern associated with the morphological variation among healthy individuals is not very clear. Gene expression complements information that is difficult to detect by visual inspection alone, and a wealth of gene expression information has been used to understand and describe differences between tissues [6]. Quantitative analysis to discover gene sets associated with nuclear morphological characteristics of healthy breast cells will enable the discovery of intrinsic drivers of differences in healthy breast tissues.

The microscopic Image or whole-slide imaging (WSI) assessment has become the most commonly used tool in clinical diagnosis and prognosis worldwide. However, the manual assessment of clinical images is subjected to artificial error, pathologist variability, and low efficiency [7]. Accurate segmentation requires pathologist knowledge, and one WSI contains up to hundreds of thousands of nuclei; it is laborious to label all nuclei, let alone calculate the features for every nucleus. It is therefore sensible to deal with such tasks by using a computer vision algorithm [8,9] which can address special work on labeling nuclei and computing a large number of nucleus features.

Many studies have been constructed using the computer vision technique in cancer diagnosis, lesion detection, etc. [10,11,12], in order to provide fast, accurate, and automatic solutions for pathologists. Specifically, there are traditional digital image processing-based algorithms [13,14] and learning-base algorithms [15,16,17]; besides, several open-source software, like QuPath [18], ImageJ [19], etc., have integrated some nuclei segmentation algorithms. The majority of earlier approaches on tissue or nuclei segmentation are achieved by supervised pixel-wise classification based on color and texture features [20,21,22,23], although a few unsupervised methods have been proposed [24,25].

There are three main tissue types in the histopathological images of the breast: glandular, connective, and adipose tissue. There are obvious differences between the nuclei of these tissues, but such vast differences will conceal the meaningful differences between individuals. Based on the above reasons, tissue segmentation was added to our nuclei segmentation process. The nuclei segmentation process includes the following steps: image normalization, extraction of stain channels, glandular tissue segmentation, nuclei segmentation, declumping, and features calculation [26].

The extracted nuclei were quantified by four common features: size, intensity, shape, and distance, consistent with previous studies [27,28]. More importantly, such features are commonly used in surgical pathological diagnosis and therefore have applications for humans. Similar to disease classification, healthy tissues can be divided into several groups according to their nuclear features.

To complement the histological images, global patterns of gene expression could demonstrate individual variations on the molecular level. Gene expression has been used to characterize cellular differences between tissue, disease phenotypes, and molecular subtypes [29,30,31]. The current association studies have mainly focused on finding connections between gene expression levels and disease phenotypes [32,33,34], while the population variation in healthy tissue associated with genetic variants has also been reported [35]. Although the GTEx consortium had comprehensively studied gene expression and gene regulation in healthy tissues [35], there is no joint analysis of image features and gene expression. In this study, we are more interested in extracting nucleus features derived from H&E-stained images of healthy breast tissue and identifying genes associated with the imaging features. We performed pathway analysis on the gene sets we found, and the results indicated that the gene sets we found were involved in immune processes related to leukocytes, cell killing, and T cells, and in the activation of serine-type endopeptidase and serine-type peptidase.

This paper is structured as follows. First, we introduce the basic theory and methodology for both imaging and genomic analysis, as well as a brief description of the study cohort. Next, we apply our framework to the GTEx dataset with histological images and gene expression levels on paired samples. We demonstrate the population variation in histological images of healthy breast tissue and discover specific genes that correlate with image features. Finally, we present biological pathways using feature-specific gene sets showing the biological significance of these genotype-phenotype associations.

## 2. Materials and Methods

### 2.1. Study Cohort

The high-resolution whole-slide images (WSIs) and the RNA-seq data were downloaded from the GTEx Portal. The RNA-Seq (GTEx Analysis V8) sample libraries had been prepared using the Illumina TruSeq Kit and paired-end sequencing was performed on the Illumina HiSeq2000. The gene read counts standardized by Transcripts Per Million (TPM) were downloaded for this study. The images of breast-mammary tissue in the GTEx database were sampled from the central breast subareolar region of the right breast, 1–2 cm under the skin surface of the nipple region. We also downloaded the sample annotation file, which documented which organization the sample came from. Breast-mammary tissue samples with both histopathology images and gene expression were screened out by sample ID matching and annotation file. All data sources can be found in the “Data Availability Statement”.

A total of 54,592 genes and 456 WSIs from 456 samples were used for this research as the study cohort. A more detailed description of data collection and preparation is referred to the original study [36].

### 2.2. Image Data Processiong

The WSIs were in SVS format with a resolution of 0.5 μm/pixel. Errors caused by the heterogeneity of nuclei in different tissue were inevitably introduced when we analyzed all the nuclei of the entire SVS. Therefore, only glandular tissue was considered in this study. Glandular tissue segmentation was performed on the lower-resolution (16 μm/pixel) images.

Every pixel in the *R*, *G*, and *B* channels were characterized by three local-robust statistics [37,38]: Median; Med(x); Inter-Quartile Range, (IQR(x)); and Median Absolute Deviation (Med(x)) [39]. Here, we define the feature vector for every pixel f(x) as:

(1) $$f\left(x\right)={\left(f_{R},f_{G},f_{B}\right)}^{T}\in R^{9},$$

(2) $$f_{R}=\left(\mathrm{Med}\left(x\right),\mathrm{IQR}\left(x\right),\mathrm{MAD}\left(x\right)\right)\in R^{3},$$

The *f_R_*, *f_G_*, *f_B_* is the feature vector that calculates on channels *R*, *G*, and *B*, respectively. The 9-dimensions vectors *f(x)* represented one pixel, then passed to the k-means algorithm. Glandular tissue areas were entered into the nuclei segmentation pipeline while glandular tissue mask images were scaled to the original size.

The nuclei were only extracted from glandular tissue. WSIs and corresponding glandular tissue mask images were cropped into 4096 × 4096-pixel tiles. The color and brightness of the 4096 × 4096 images were first normalized by histogram equalization in the L channel of the CIELAB color space. Then, the color unmixing technique [40] was applied for the hematoxylin component extraction to highlight the chromatin material and for subsequent nuclei segmentation. Next, the Otsu method with the Otsu ratio threshold equal to 1 was performed for finding the initial locations of the nuclei contours. The initial contours were optimized to be smoother and more accurate by level-set-based contour evolution algorithms [41]. There were some regions in which multiple nuclei clumped without clear separation. In such cases, the mean-shift algorithm was applied to separate the chunk into individual nucleui [42].

Once the nuclei in the WSI were segmented, a rich set of imaging features describing the extracted nuclei was calculated. These features summarized the size, intensity, and geometric shape characteristics of each detected nucleus, and some of them are essential for downstream combination analysis. Implementations of these features were part of the ITK toolkit [43]. It is neither feasible nor consistent to list the features of all nuclei for follow-up analysis. Instead, we used a list of aggregating statistics—mean value, standard deviation, and median value—to summarize them for each WSI.

In addition to each individual nucleus, the spatial distribution of nuclei in the tissue was also found crucial in some pattern recognition tasks [44]. After we acquired the spatial centroids of detected nuclei, we generated Voronoi diagrams, Delaunay triangulation, and minimum spanning trees from these locations. Then, neighboring nuclei counts within a given radius were calculated to quantify the spatial arrangement of nuclei. The final feature values were aggregated with the local feature values of the entire WSI. All features used in this study are listed in Supplementary Table S1.

### 2.3. Unsupervised Analysis of Breast Images Using Nucleus Features

**Figure 1:**
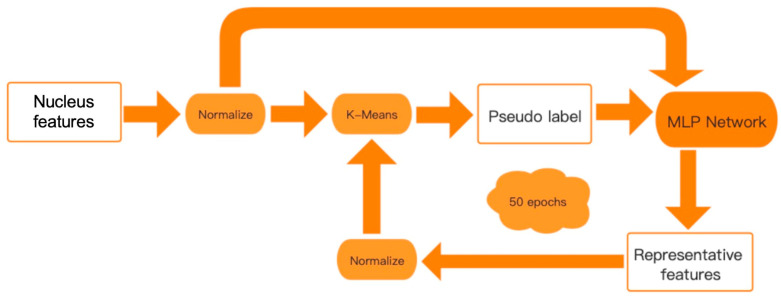
Pipeline of the image processing. Two parts are consisted in our method: representative features are extracted by multilayer perceptron, and the clustering is performed by k-means. The nucleus features were firstly used to produce the pseudo label by k-means for training the MLP Network, and the representative features were used to produce the pseudo label again. The framework iterated 50 epochs until convergence.

A deep clustering procedure [45,46] was used in this study, which contains a multilayer perceptron (MLP) network module for extracting the representative features and a k-means module for clustering samples [47]; the details are shown in Figure 1.

To determine the appropriate number of clusters, we applied the k-means clustering algorithm with k = 1, 2, …, 14 and calculated the sum of the squared errors (SSE). As shown in Supplementary Figure S1, the change in slope of the SSE curve is lowering when k > 4, so we chose k = 4, at the elbow of the curve. We also validated the k-means clustering results with k = 3, 4, 5 by UMAP (Supplementary Figure S2). The UMAP plot indicates well separation in the four clusters but not in the three or five clusters.

There are 3 hidden layers in the MLP network for features extraction, in which the number of output units is 100, 100, and 50, respectively, and each output was activated by Rectified Linear Unit (ReLu) function. The final activation output went through a linear classifier by softmax, and the cross-entropy was used as the loss function. In each iteration, the MLP network was trained in 500 epochs by the Adam optimization algorithm. In this study, the input data of the MLP network and the final activation output for clustering were normalized with the target mean and standard deviation of 0.0 and 1.0, respectively. The iterative process continues 50 times for convergence.

### 2.4. Identification of Feature-Specific Genes

The analyzed dataset was filtered (retaining only genes with an estimated expression TPM > 0 in more than 10% of samples) before doing statistical analysis. The differential expression analysis algorithm, originally described in [48], underwent a minor adaptation to fit our four clusters. Genes we found were over-expressed in a single cluster, while their expression was not statistically different among the remaining clusters. While the traditional algorithm such as DEseq2 [49] only considers the comparison between one group and the others, comparing one cluster against the rest does not guarantee that the selected genes are only specific to one cluster.

Two statistics were calculated for hypothetical testing: a robust t statistic (T1) and a chi-square statistic (T2) for each gene, and the cluster-specific genes were screened out by T1 and T2. T1 was used to determine if a gene over-expresses in a special subtype compared to the others, and T2 was used to determine if there is no significant difference among the others. To find the genes that meet the conditions described above, the T1 must be large enough to guarantee that there is a significant difference of the expression in one cluster compared to the others, and the T2 must be small to ensure that there is no significant difference in the expression among the other clusters.

To obtain statistical significance, we computed *p*-values from T1 and T2, then accounted for multiple tests using the false discovery rate (FDR) [50]. In this study, we set the thresholds of T1-based FDR < 0.01 and T2-based FDR > 0.1.

## 3. Results

### 3.1. Image-Genetic Joint Analysis Pipeline

**Figure 2:**
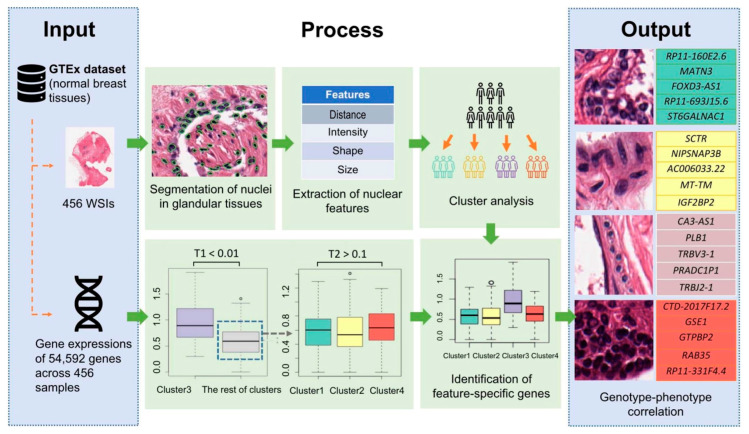
Pipeline of the association analysis between image-based features and RNA-expression profiles of healthy breast tissue. A total of 456 H&E images and the corresponding RNA-seq data from the GTEx database were included in the analysis. Sixty-five intensity and texture features of nuclei in glandular tissue were computed and then classified into four clusters. We discovered 1447 genes specific to single clusters, and the top 5 genes of each cluster are shown in the output panel. The circles in the boxplot represent outliers in the data.

The whole joint pipeline for the image-and-gene analysis could be described as follows: In the pre-processing of the WSI, we obtained a multiscale stack of images including the low-resolution one used to extract the glandular region. Then, we extracted the nuclei and calculated the statistics of the nuclei’s morphological characteristics. Finally, we performed the joint analysis of nucleus characteristics and RNA-expression to find the gene sets that are associated with specific nucleus features. The pipeline of the image-feature-specific genes discovery analysis is demonstrated in Figure 2.

### 3.2. Glandular Tissue Segmentation

The inherent differences of nucleus features in different tissues have a huge impact on cluster analysis, e.g., nuclei from areas of adipose tissue are small and elongated, so true biological differences between individuals will overwhelmed by such effects. Also, the nucleus features in glandular tissue are most likely associated with breast diseases.

**Figure 3:**
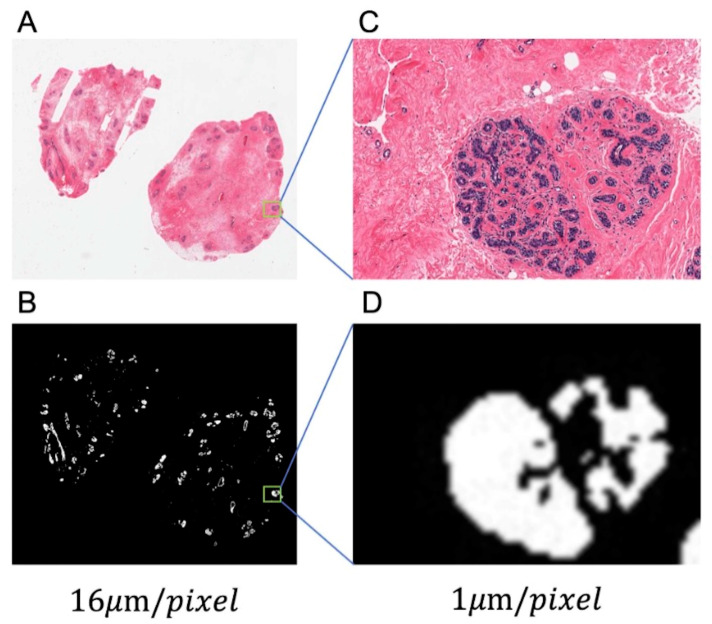
One example of glandular tissue segmentation. (**A**) The standard image at its lowest resolution to show the global view. (**B**) The corresponding global mask image in which the white part represents the glandular tissue. (**C**,**D**) The corresponding zoomed-in version.

Each image had been segmented into background, adipose tissue, glandular tissue, and connective tissue. To confirm which cluster represents the glandular tissue, we picked one standard image (where the glandular tissue is segmented precisely with pathologist verification) and used centroid of the glandular tissue cluster as the reference. For each image, the cluster whose centroid is closest to the reference was considered to be glandular tissue. The standard image and the corresponding mask image are shown in Figure 3. The top half of Figure 3 is the WSI in a resolution of 16 μm/pixel (A) and 1 μm/pixel (C), whereas the bottom half part of Figure 3 indicates the binary mask for the glandular region in the WSI.

Following the method in Section 2.2, the glandular regions were segmented at low resolution. After that, the segmentation mask was resized to the original resolution for guiding the nucleus segmentation.

### 3.3. Nuclei Segmentation

**Figure 4:**
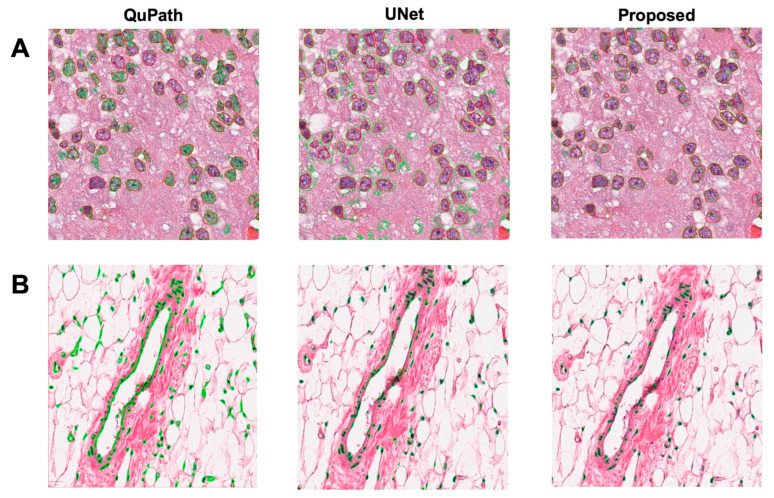
Two example results of three nucleus segmentation methods for (**A**) challenge data and (**B**) GTEx data. Contour colors: red (ground truth), green (algorithm).

The performance of the proposed nucleus segmentation method had been compared to two state-of-the-art nucleus segmentation programs, i.e., a UNet-based approach [51] and QuPath (version 0.3.2) [18]. Due to the lack of nucleus annotations in GTEx histopathology images, algorithm comparisons were performed on a collection of images from MICCAI, Kaggle, and ISBI challenges [52,53,54,55]. A total of 96 images with ground truth labels were split into training, validation, and test sets, with 82, 4, and 3 images, respectively. Dice Similarity Coefficient (DSC), Intersection over Union (IoU) and Hausdorff Distance (HD) were calculated to evaluate the performance of the algorithms. As shown in Table 1, our proposed method outperforms other compared methods, i.e., DSC is 10% better than QuPath and 2% better than UNet, IoU is 18% better than QuPath and 3% better than UNet, and Average HD is 53% better than QuPath and 0.7% better than UNet. Figure 4 shows two examples of three nucleus segmentations for both challenge data and GTEx mammary data. The UNet and QuPath show various degrees of clumping, while the proposed algorithm explicitly declumped the chunk into individual nuclei.

### 3.4. Classification of Image Features

**Figure 5:**
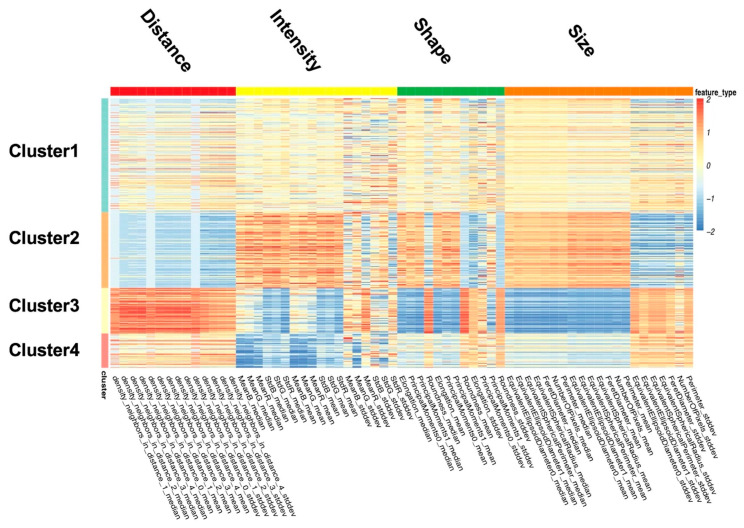
Heat map of clustering results for nucleus features. Each row represents a sample and each column represents a nucleus feature. Feature scores in this heat map were normalized.

Since GTEx samples are from healthy donors, there is no pathological classification associated with them. To discover the stratification of nuclear features, we use a deep clustering method to divide all samples into four clusters according to all nuclear features, as described in Section 2.3. Furthermore, the heat map shown in Figure 5 describes the features of each cluster. Each row represents one sample, and each column represents one feature. The numerical values in Figure 5 were z-scores normalized to mean = 0 and variance = 1. Here we list the characteristics of each cluster:

- Cluster 1: All nuclear features are close to the sample mean.
- Cluster 2: The nuclei in this cluster are large, irregular, long, and dark, with the most uneven color distribution. The distance between the nuclei is small.
- Cluster 3: The nuclei in this cluster are small and round, with uniform color distribution. The distances among the nuclei are large.
- Cluster 4: The nuclei in this cluster appear to be quite dark.

**Figure 6:**
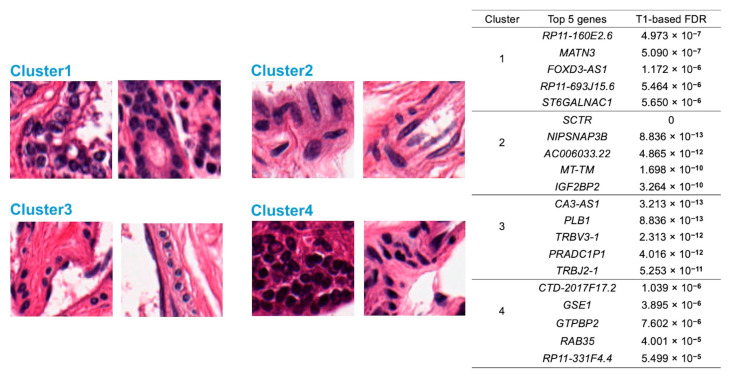
Illustration of images sampled from those with the most extreme image features in each cluster. Correspondingly, the top five feature-specific genes that are most highly expressed in each cluster are also shown.

Figure 6 shows images of two samples from each cluster.

### 3.5. Discovery of Feature-Specific Genes

The classification of GTEx samples in Section 3.3 was used for the identification of feature-specific genes where expressions are significantly higher in one particular cluster than the others. Figure 6 shows two examples of nuclei and the top five specific genes for each cluster.

**Figure 7:**
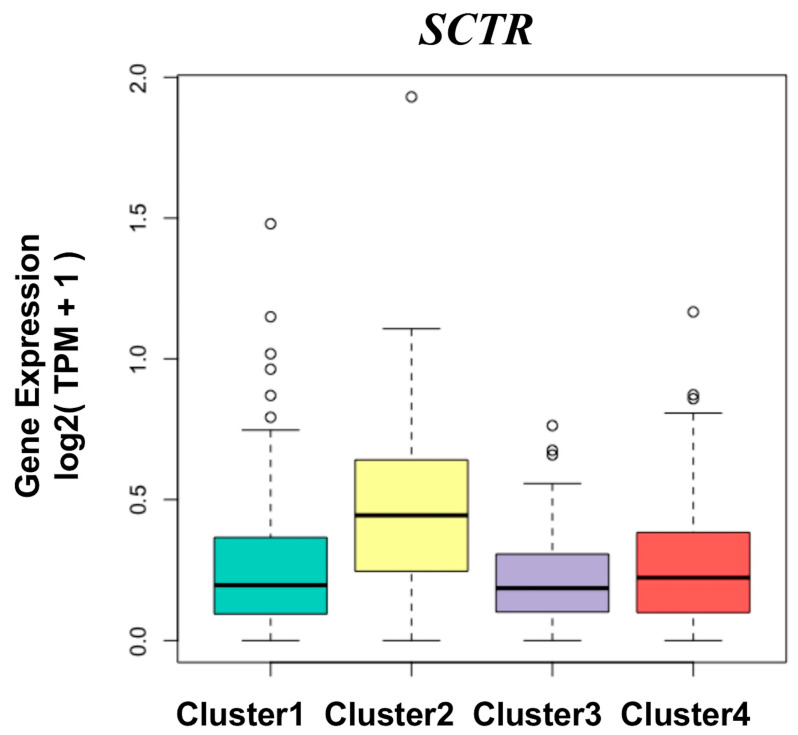
The gene-level expression distribution of the *SCTR* gene across four clusters, where the value of the ordinate is log2(TPM+1). The circles in the boxplot represent outliers in the data.

**Figure 8:**
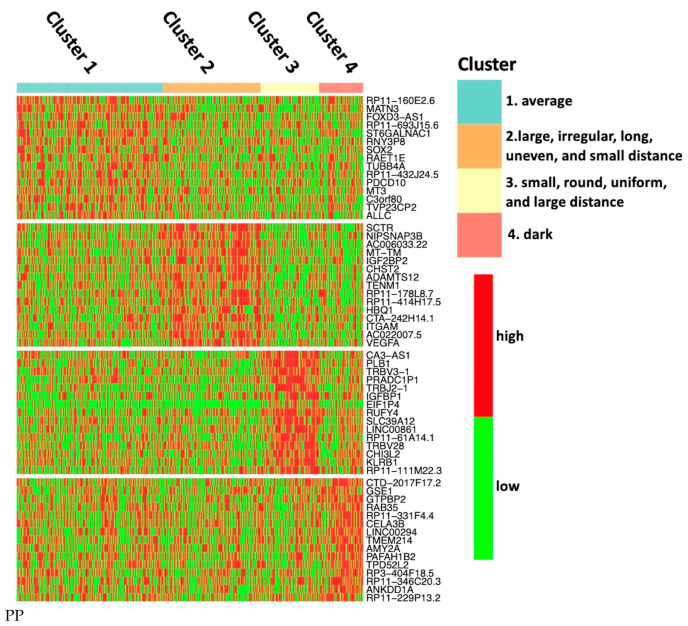
Color-map of the top 15 feature-specific genes from each cluster. Each row represents a gene, and each column represents a sample. Red and green indicate a gene’s mRNA expression level above and below its median expression level across all samples, respectively. The genes in each cluster were ordered by *p*-value from bottom to top.

As an example of feature-specific genes, Figure 7 shows the boxplot of gene-level mRNA expressions of *SCTR*, a top-ranking gene specific to Cluster 2. It is highly expressed in Cluster 2 and lowly expressed in the rest of the clusters. The cluster-specific genes were selected following two criteria: T1 less than 0.01 and T2 greater than 0.1, which ensures that the expression of cluster-specific genes are higher in one specific cluster than the others, while there is no significant difference in the others [56]. A total of 141, 359, 740, and 207 genes were considered as the feature-specific genes for each cluster, respectively. After being ranked by an ascending order of T1, the top 25 feature-specific genes are shown in Supplementary Table S4, and all genes are shown in the CSV table in our Supplementary Material. The expression of the first gene in each cluster is displayed in Supplementary Figure S3. Figure 8 is the color map of the top 15 genes in each group, in which each row represents one gene and each column represents one sample. In Figure 8, there are four red blocks, which indicates that the expression of the feature-specific gene is higher in one specific cluster than the others. In specific, Clusters 2, 3, and 4 are explicit, while the first block is not.

*SCTR*, the top gene in Cluster 2, is a protein-encoding gene that encodes the G protein-coupled receptor that binds the secretin. Cluster 2 is the cluster with the most distinctive characteristics. The dysregulation of *SCTR* had been reported, which relates to a few cancers. Onori et al. [57] had reported that the secretin inhibits the cholangiocarcinoma growth via dysregulation of the cAMP-dependent signaling mechanisms of the secretin receptor, and the modulation of *SCTR* expression might behave as a tool to treat cholangiocarcinoma. Li et al. [58] had reported that hypermethylation at the CpG island of *SCRT* is the diagnostic biomarker of colorectal cancer and its precursor lesions. Concerning the effect of *SCRT* on breast tissue, Kang et al. [59] had reported that *SCTR* suppresses the proliferation of normal breast cells, while the downregulation by promoter methylation stimulates the proliferation and migration of cancer cells. *IGF2BP2* (Insulin-Like Growth Factor 2 MRNA Binding Protein 2), the Cluster 2-specific gene, is a protein-encoding gene, which encodes the protein binding the 5’ UTR of insulin-like growth factor 2 (IGF2) mRNA and regulating its translation. *IGF2BP2* also relates to several cancers, including liver, pancreatic, breast, and so on. McMullen et al. [60] had reported that *IGF2BP2* is significantly upregulated in metaplastic carcinoma of the breast. Kim et al. [61] had reported that insulin-like growth factor 2 mRNA binding protein 2 and 3 are upregulating in triple-negative breast cancer and cooperating to promote the migration and invasion of cancer cells.

*CA3-AS1* (CA3 Antisense RNA 1), the top-rank gene of Cluster 3, is a long non-coding RNA (lncRNA). Cluster 3 is a cluster with opposite characteristics to Cluster 2. Zhang et al. [62,63] had reported that the overexpression of *CA3-AS1* which locates in the cytoplasm can suppress the proliferation and invasion of colorectal cancer cells by binding to miR-93.

### 3.6. Pathway Enrichment Analysis

We performed pathway enrichment analysis on a subset of signature-specific genes for each cluster. The analysis based on the Reactome [64] database was carried out on the website “https://reactome.org/ (accessed on 14 December 2022)”, and the analysis based on the KEGG [65] and Gene Ontology (GO) [66] databases was performed using the enrichKEGG and enrichGO functions of the R package clusterProfiler v3.18.1 (*p*-value was adjusted by the Benjaminiand Hochberg method).

**Figure 9:**
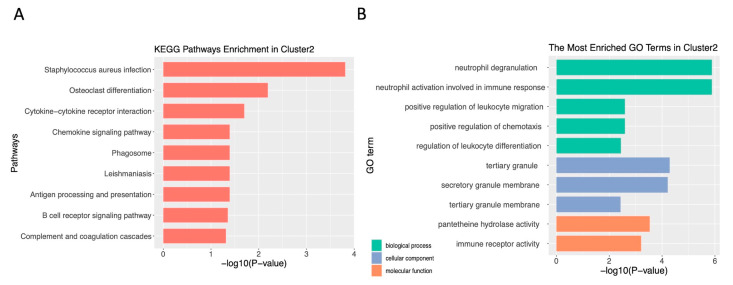
Visualization of enrichment analysis. (**A**) Bar graph of significant pathways in KEGG analysis. (**B**) Bar graph of significant pathways in GO analysis.

The significant pathways with *p*-values less than 0.05 for each cluster in each database, as well as relative statistics, are listed in Supplementary Table S5. For instance, the most significant pathway related to Cluster 2 in GO (adjusted *p*-value = 2.32 × 10^−2^) and in KEGG (adjusted *p*-value = 1.54 × 10^−4^) analysis is “neutrophil degranulation” (GO:0043312), and “Staphylococcus aureus infection” (hsa05150). The KEGG and GO analysis results of Cluster 2 are summarized in Figure 9; the results of the other three clusters are summarized in Supplementary Figures S4–S6. The most significant pathway related to Cluster 2 (Entities *p*-value = 8.20 × 10^−4^) in Reactome analysis is “Neutrophil degranulation.” Neutrophil is the most abundant leukocyte and it plays a very important role in the nonspecific immune system. Neutrophil-like populations are recognized as having an important role in cancer development [67], Moreover, neutrophil granule proteins may mediate tumor cell metastasis to different tissues and develop into different cell types [68]. We also used the top 25 feature-specific genes in each cluster to carry out the pathway analysis by the Reactome database. The significant pathways with *p*-values less than 0.05 for each cluster and relative statistics are listed in Supplementary Table S6.

## 4. Discussion

In this study, we proposed a joint analysis framework for paired histopathological images and gene expressions of healthy breast tissue. This analytical framework quantitatively computed the morphological features of the nuclei and divided samples into four well-characterized clusters based on nuclear features. Finally, we identified a set of feature-specific genes that are associated with healthy breast tissue growth and breast disease development, including the proliferation of normal breast cells, the development of breast lesions, and the metastasis and proliferation of cancer cells. We have provided a comprehensive view of the transcriptomic landscape of molecular feature-specific RNA expression of breast tissue.

The proposed analytical model is able to identify phenotypic differences across healthy breast tissues based on the sizes and color depths of nuclei. Compared to the healthy tissue, diseased tissue has a high degree of heterogeneity caused by disease type, disease grade, and so on. Such multiple factors would jointly affect the phenotypic characteristics of diseased tissue. To avoid the manifold influence from disease and tissue-type, our study is focused on the healthy glandular tissues of the breast. This ensures differences between individuals would not be disturbed by redundant factors.

In order to verify the biological significance of four feature-specific gene sets, we performed pathway analysis. For example, the specific genes of Cluster 2 are closely related to immune regulation: “neutrophil degranulation” (GO:0043312), “neutrophil activation involved in immune response” (GO:0002283), “B-cell receptor signaling pathway” (hsa04662) and “chemokine receptors bind chemokines.” The neutrophil-to-lymphocyte ratio plays an important role in breast cancer prognosis [69]. Chemokines, chemokine rectors, and neutrophil granule proteins are involved in tumor metastasis [68,70].

Some of the genes in the discovery gene sets are associated with breast cancer; further explorations could be made based on the identified feature-specific biomarkers. For instance, two feature-specific genes of Cluster 3 (UBE2C and NDC80) are also in the set of PAM50 [71]. UBE2C (Ubiquitin Conjugating Enzyme E2C) is a Protein Coding gene that encodes a member of the E2 ubiquitin-conjugating enzyme family. Its high expression relates to the poor prognosis in high-risk breast cancer [72]. It is also a direct target of miR196-a, which promotes cell proliferation in breast cancer [73]. NDC80 is a protein Coding gene that encodes the NDC80 kinetochore complex components (NUF2). It may be involved in preneoplastic processes, as it is detected in benign breast tumors [74]. Xu et al. [75] had reported that NUF2 is overexpressed in breast cancer and significantly connected to multiple pathological features and prognosis of breast cancer. MDM2 from Cluster 4 is a cancer-related gene that encodes a nucleus-localized E3 ubiquitin ligase; such protein can promote tumor formation by targeting tumor suppressor proteins, e.g., p53, for proteasomal degradation. The study of Opoku et al. [76] shows that MDM2 might be associated with aggressive biological behavior in breast cancer. It could be a biomarker implying the poor Overall Survival (OS) and Progression-Free Survival (PFS) in luminal breast cancer [77].

Although we intuitively describe the concrete features of the nucleus, some abstract features could be lost. These features may describe the images in more detail. In future study, we will combine abstract features such as deep features [78] to further supplement image descriptions, explore genes related to these features, and conduct joint analysis with our findings.

All of our results suggest that the gene-expression profiles not only characterize the molecular subtypes of diseases, but also provide an explanation of imaging phenotypes. This study reveals a link between genotype at the nanometer scale and nuclear phenotype at the micrometer scale in healthy breast tissue. We found a stratification of nuclear phenotypes and associated gene sets in healthy tissues, while accounting for heterogeneity in diseased tissue and differences across tissue types. These findings provide novel view and biomarkers of healthy breast tissue.

## 5. Conclusions

In this study, we developed a computational framework for paired histological images and RNA expressions to identify feature-specific genes that are associated with nucleus morphology. The framework had been applied on 456 paired samples from GTEx with both histological images and RNA-seq data. The analysis shows strong evidence in support of the unsupervised deep learning approach to extract histological image features and the quasi-Poisson based method to identify feature-specific genes. The proposed analysis unveils the individual variation and helps to understand how regulation of gene expression in tissue related to tissue morphology. Ongoing studies include extending the proposed pipeline to more tissue types in the GTEx dataset.

## Figures and Tables

**Table 1:** The performance of nucleus segmentation methods. The proposed method outperforms the other methods.

	DSC (mean ± std)	IoU (mean ± std)	Average HD (mean ± std)
QuPath	0.7002 ± 0.0904	0.5451 ± 0.1006	2.8108 ± 2.0811
UNet	0.7592 ± 0.0983	0.6204 ± 0.1187	1.3044 ± 0.7208
Proposed	0.7797 ± 0.0525	0.6416 ± 0.0691	1.2942 ± 0.6634

## Data Availability

RNA-sequencing data “https://storage.googleapis.com/gtex_analysis_v8/rna_seq_data/GTEx_Analysis_2017-06-05_v8_RNASeQCv1.1.9_gene_tpm.gct.gz (accessed on 14 October 2021)”, tissue slide images for the mammary samples “https://gtexportal.org/home/histologyPage (accessed on 14 October 2021)” and sample annotations file “https://storage.googleapis.com/gtex_analysis_v8/annotations/GTEx_Analysis_v8_Annotations_SampleAttributesDS.txt (accessed on 14 October 2021)” were downloaded from the GTEx Portal “https://www.gtexportal.org/home (accessed on 14 October 2021)”. Details of the data collection and preparation can be found in the original studies [36]. The tissue slide images for validating the performance of nucleus segmentation algorithm were downloaded from 4 datasets: Medical Image Computing C.A.I.S. Miccai2016 “http://www.miccai2016.org/en (accessed on 14th October 2021)”, Isbi2015 “https://biomedicalimaging.org/2015/program/isbi-challenges (accessed on 14 October 2021)”, Medical Image Computing C.A.I.S. Miccai2015 “https://warwick.ac.uk/fac/cross_fac/tia/data/glascontest (accessed on 14 October 2021)” and Kaggle2018 “https://www.kaggle.com/c/data-science-bowl-2018 (accessed on 14 October 2021)”. The code for discovering the feature-specific genes in this manuscript is publicly available at [48], the code for image features and specific genes analysis is publicly available at “https://github.com/liangjianwen01/GTExMammary (created on 26 December 2022)”, the remaining codes can be available by contacting the corresponding author.

## Supplementary Materials

The following supporting information can be downloaded at: https://www.mdpi.com/article/10.3390/s23031432/s1, Supplementary file: Figure S1: SSE plot and SSE Difference plot for k-means; Figure S2: UMAP plot for k-means with k = 3, 4, 5; Figure S3: Boxplot for expression of the first gene in each cluster; Figure S4: The KEGG analysis results of Cluster 1; Figure S5: The KEGG and GO analysis results of Cluster 3; Figure S6: The KEGG and GO analysis results of Cluster 4; Table S1: A table of explanations of image features.; Table S2: A table of top 25 genes specific to each cluster; Table S3: A table of pathways discovered by the specific genes of each cluster by Reactome, which *p*-value is less than 0.05; Table S4: A table of pathways discovered by the specific genes of each cluster by KEGG, which *p*-value is less than 0.05; Table S5: A table of pathways discovered by the specific genes of each cluster by GO, which *p*-value is less than 0.05; Table S6: A table of pathways discovered by the top 25 genes of each cluster by Reactome, which *p*-value is less than 0.05; Specific_Genes.csv: A CSV table of all specific genes of 4 clusters.
