# Supplementary material for: A Comprehensive Landscape of Imaging Feature-Associated RNA Expression Profiles in Human Breast Tissue
Source: Sensors (Basel). 2023 Jan 28;23(3):1432. doi: 10.3390/s23031432 (PMC9921444; doi:10.3390/s23031432)
Supplement: Supplementary file 1 [file sensors-23-01432-s001.zip › Supplementary_Material.pdf]

Supplementary Material

1 Supplementary Figures and Tables

1.1 Supplementary Figures

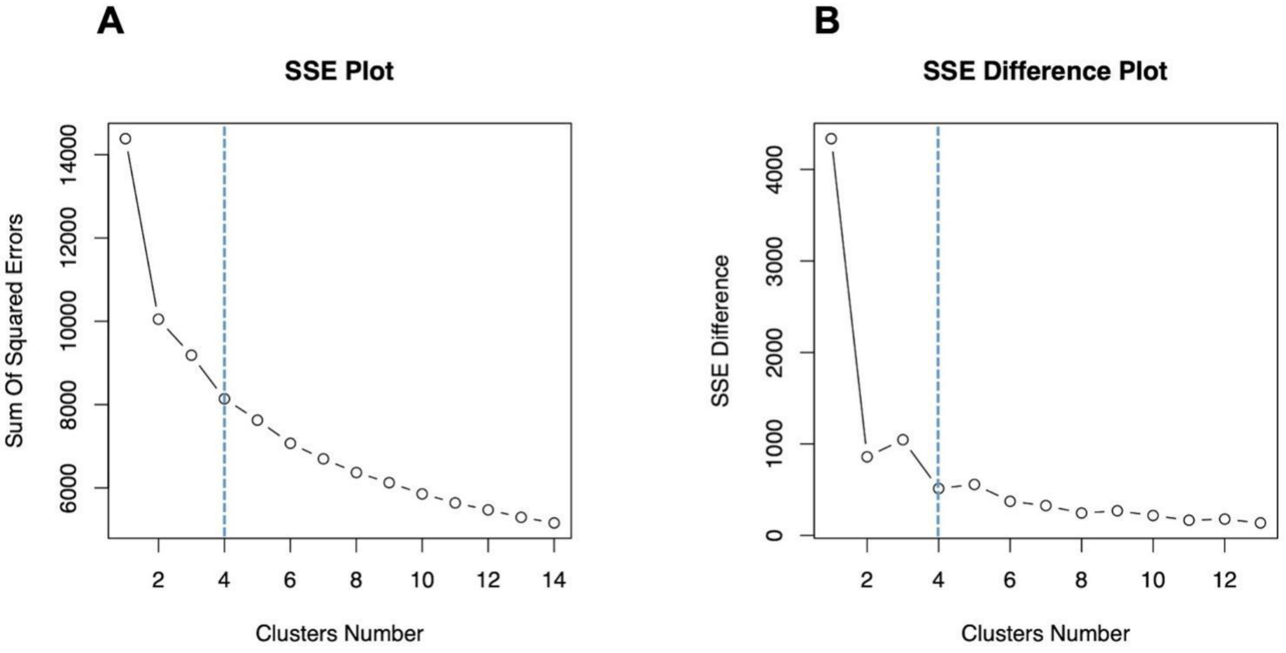

FIGURE S1 Plots of sum of squared errors (A) and SSE difference (B) of k-means with k=1,2,...,14. We choose k=4 at the elbow of the curve.

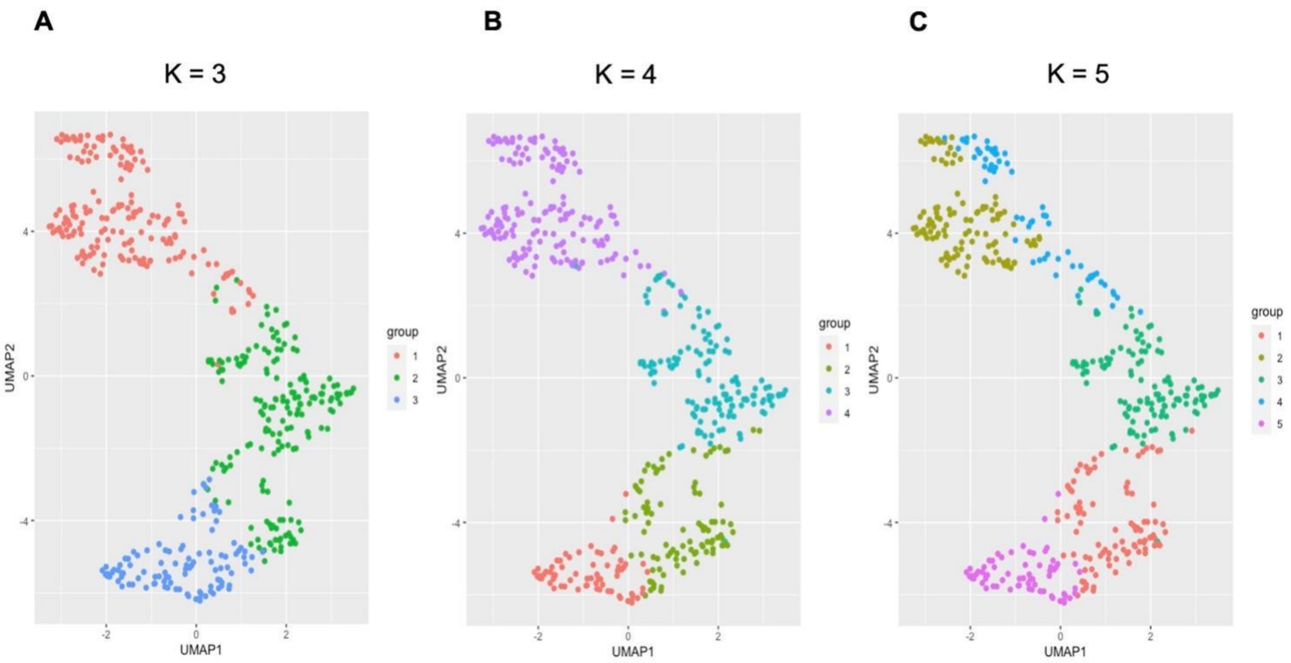

FIGURE S2 The UMAP plot of k-means clustering results with k=3, 4, 5.

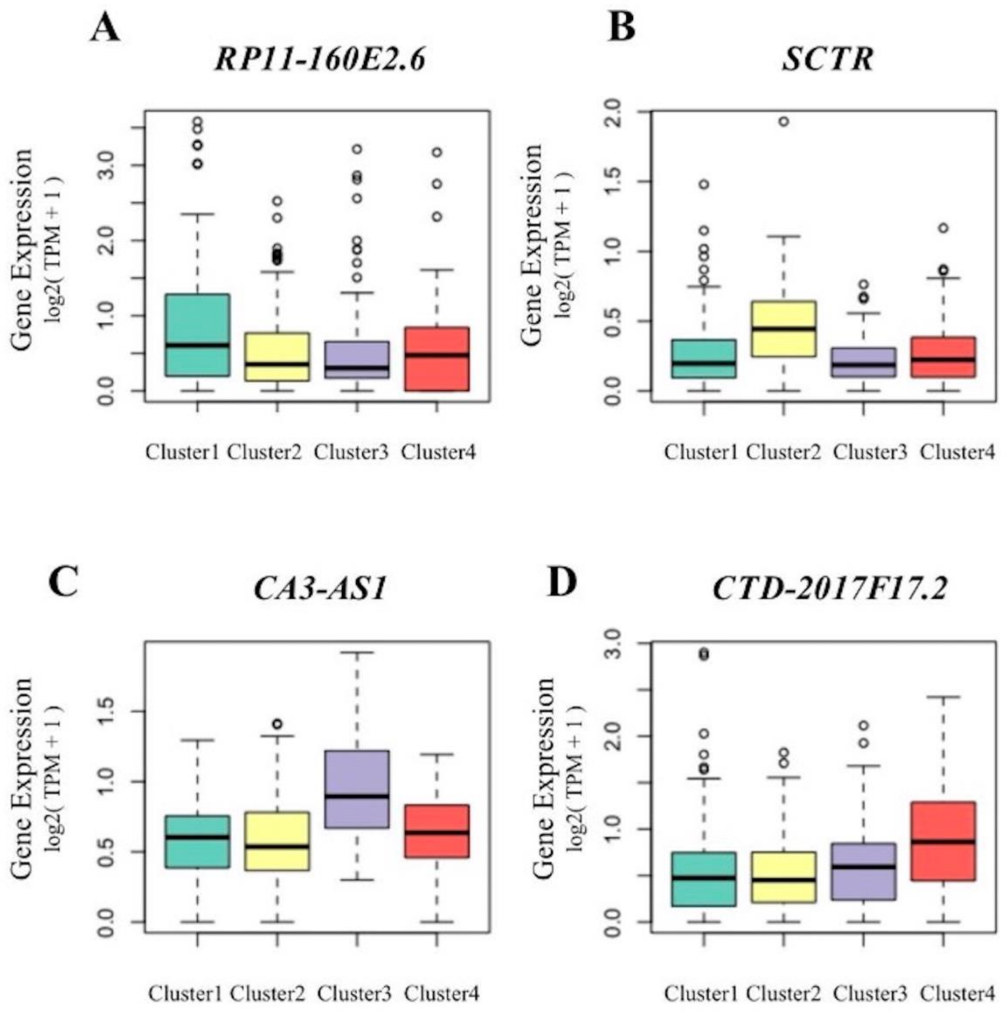

FIGURE S3 The boxplot of the top genes which specify to Cluster 1 (A), Cluster 2 (B), Cluster 3 (C), Cluster 4 (D) respectively. The circles in the boxplot represent outliers in the data.

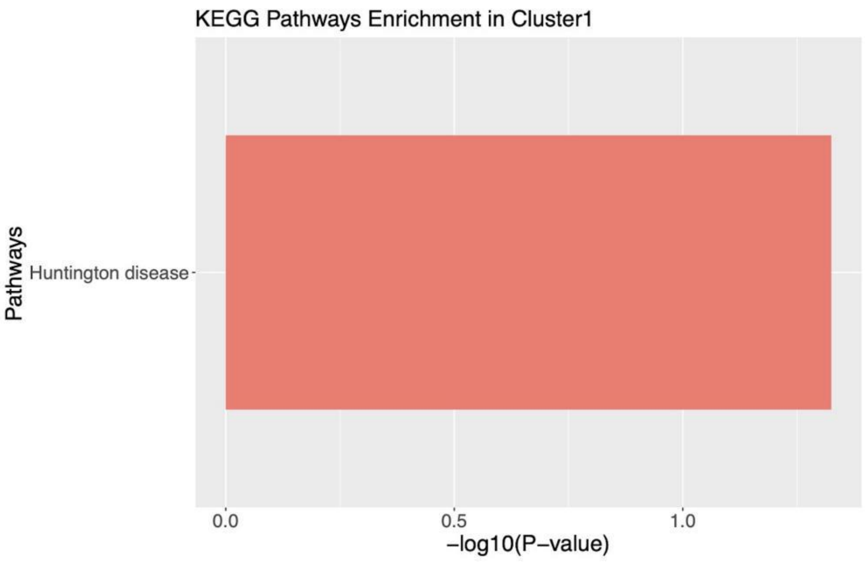

FIGURE S4 Visualization of enrichment analysis in Cluster1. Bar graph of significant pathways in KEGG analysis.

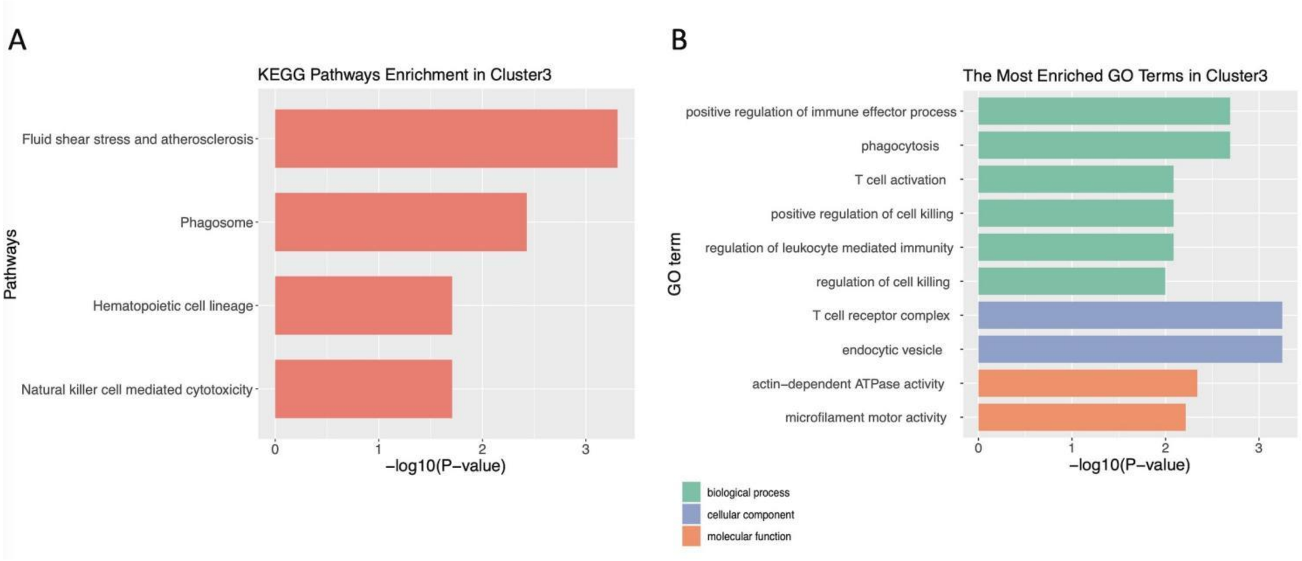

FIGURE S5 Visualization of enrichment analysis in Cluster3. (A) Bar graph of significant pathways in KEGG analysis (B) Bar graph of significant pathways in GO analysis.

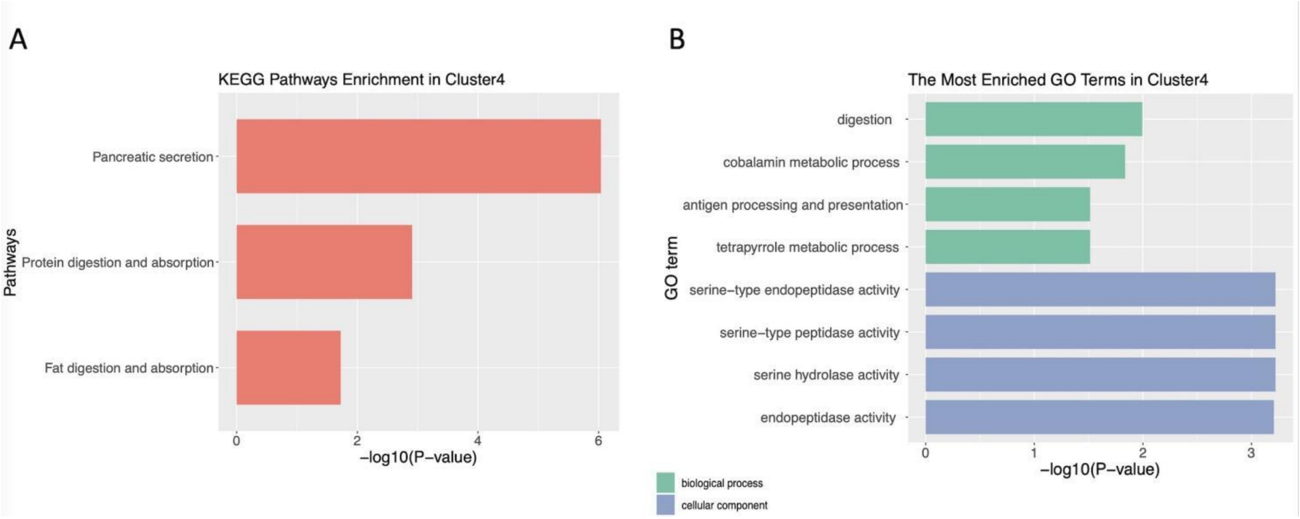

FIGURE S6 Visualization of enrichment analysis in Cluster4. (A) Bar graph of significant pathways in KEGG analysis (B) Bar graph of significant pathways in GO analysis.

1.2 Supplementary Tables

Table S1 Explanations of image features.

Feature Type	Feature Name	Feature Explanations
Shape	Centroid	Centroid of the object
	Circularity/Roundness	Quantify how close the object should be to a true circle. It is the ratio of the area of the equivalent hypersphere to the measured area of the object.
	Elongation	Ratio of the longest physical size of the object on one dimension and its smallest physical size
	EquivalentEllipsoidDiameter, EquivalentEllipsoidDiameter0, EquivalentEllipsoidDiameter1	Diameters of the ellipsoid of the same size and the same ratio on all the axes of the object
	EquivalentSphericalPerimeter	Equivalent perimeter of the hypersphere of the same size of the object

	EquivalentSphericalRadius	Equivalent radius of the hypersphere of the same size of the object
	FeretDiameter	Diameter in physical units of the sphere which include all the object
	Flatness	Degree to which the object approximates the hyperplane
	MajorAxis, MinorAxis	Longest/shortest diameter of the object
	Perimeter	Perimeter of the object
	PerimeterOnBorderRatio	Ratio of perimeter of object which are on the border of the image to the whole perimeter
	PrincipalAxes	Principle axes of the object
	PrincipalMoments, PrincipalMoments0, PrincipalMoments1	Principle moments of the object
Size	AreaInPixels, NumberOfPixels	Area of the object in terms of number of pixels
	NumberOfPixelsOnBorder	Number of pixels of object which are on the border of the image
	PerimeterOnBorder	Perimeter of object which are on the border of the image
	PhysicalSize	Size of the object in physical unit
Texture/Intensity	Intensity_[Mean/Max/Min/Std] or Mean[R/G/B] or Std[R/G/B]	Mean/Max/Min/Std statistics of the intensity values in the object of R/G/B channels

	IntensityKurtosis	Kurtosis of the intensity values in the object of R/G/B channels
	IntensityEnergy, IntensityEntropy	Energy and entropy of the intensity values in the object of R/G/B channels
	GradientEnergy, GradientEntropy	Energy and entropy of the intensity values in the gradient image of object of R/G/B channels
	GradientMean, GradientStd, GradientSkewness, GradientKurtosis	Mean/Std/Skewness/Kurtosis of the intensity values in the gradient image of object of R/G/B channels
	CannyMean	Average of canny value of the object
	CannyNonZero	Number of pixel with nonzero canny value of the object
	cytoCannyMean	Average of canny value of the cytoplasm
	cytoCannyNonZero	Number of pixel with nonzero canny value of the cytoplasm
	cytoGradientEnergy, cytoGradientEntropy	Energy and entropy of the intensity values in the gradient image of cytoplasm of R/G/B channels
	cytoGradientMean, cytoGradientStd, cytoGradientSkewness, cytoGradientKurtosis	Mean/Std/Skewness/Kurtosis of the intensity values in the gradient image of cytoplasm of R/G/B channels
	cytoIntensityEnergy, cytoIntensityEntropy	Energy and entropy of the intensity values in the gradient image of cytoplasm of R/G/B channels
Distance	density_neighbors_in_distance_[0, 1, 2, 3, 4]	Neighbor count within given radius

Table S2 List of top 25 genes, which are ranked according to T1, specify for each cluster.

	**Genes**	**T1-based FDR**	**T2-based FDR**
Cluster1	*RP11-160E2.6*	4.970E-07	8.002E-01
	MATN3	5.090E-07	6.586E-01
	FOXD3-AS1	1.170E-06	6.229E-01
	RP11-693J15.6	5.460E-06	6.195E-01
	ST6GALNAC1	5.650E-06	7.396E-01
	RNY3P8	1.300E-05	6.195E-01
	SOX2	1.370E-05	8.288E-01
	RAET1E	3.280E-05	6.195E-01
	TUBB4A	3.280E-05	7.567E-01
	RP11-432J24.5	5.030E-05	6.210E-01
	PDCD10	5.520E-05	6.195E-01
	MT3	6.870E-05	6.195E-01
	C3orf80	8.060E-05	8.755E-01
	TVP23CP2	1.339E-04	7.766E-01
	ALLC	1.444E-04	6.195E-01
	ENY2	1.573E-04	8.204E-01

	KIRREL-IT1	1.645E-04	9.734E-01
	LINC00359	2.518E-04	6.195E-01
	RNU6-415P	2.784E-04	8.607E-01
	FRY	3.126E-04	7.375E-01
	RP11-779O18.1	3.619E-04	6.195E-01
	RP11-620J15.4	3.816E-04	9.095E-01
	NPY1R	4.436E-04	6.195E-01
	FAM153B	4.439E-04	6.249E-01
	RP11-372K14.2	5.468E-04	6.461E-01
Cluster2	*SCTR*	0.000E+00	6.195E-01
	NIPSNAP3B	8.840E-13	6.195E-01
	AC006033.22	4.870E-12	6.195E-01
	MT-TM	1.700E-10	6.247E-01
	IGF2BP2	3.260E-10	7.218E-01
	CHST2	6.640E-10	6.195E-01
	ADAMTS12	9.970E-10	6.195E-01
	TENM1	2.120E-09	6.210E-01
	RP11-178L8.7	2.390E-09	6.195E-01
	RP11-414H17.5	2.570E-09	6.308E-01

	HBQ1	3.060E-09	6.586E-01
	CTA-242H14.1	7.500E-09	8.755E-01
	ITGAM	7.500E-09	6.195E-01
	AC022007.5	9.520E-09	6.195E-01
	VEGFA	1.100E-08	6.586E-01
	CSTB	1.510E-08	6.689E-01
	SMOC1	1.730E-08	7.759E-01
	RP11-366M4.11	1.730E-08	6.960E-01
	DNAH9	1.860E-08	6.238E-01
	APOE	3.250E-08	6.443E-01
	VNN2	3.520E-08	6.195E-01
	FAM27E2	7.080E-08	6.210E-01
	CRYAB	7.290E-08	6.247E-01
	NCF4	1.070E-07	6.203E-01
	GIPR	1.070E-07	6.195E-01
Cluster3	*CA3-AS1*	3.210E-13	6.195E-01
	PLB1	8.840E-13	6.247E-01
	TRBV3-1	2.310E-12	6.244E-01
	PRADC1P1	4.020E-12	6.195E-01

	TRBJ2-1	5.250E-11	6.238E-01
	IGFBP1	1.170E-10	6.577E-01
	EIF1P4	2.120E-10	1.000E+00
	RUFY4	4.020E-10	6.195E-01
	SLC39A12	6.640E-10	6.690E-01
	LINC00861	6.960E-10	6.318E-01
	RP11-61A14.1	9.970E-10	6.195E-01
	TRBV28	1.380E-09	9.027E-01
	CHI3L2	2.000E-09	6.195E-01
	KLRB1	2.120E-09	6.195E-01
	RP11-111M22.3	2.120E-09	6.247E-01
	HIST1H2BK	2.780E-09	6.195E-01
	AC060834.2	2.930E-09	6.195E-01
	CTD-2161E19.1	3.260E-09	6.247E-01
	RP11-521D12.5	6.060E-09	6.247E-01
	DUXAP10	6.060E-09	6.229E-01
	RP11-67L3.4	7.500E-09	6.195E-01
	LL22NC03-N14H11.1	9.520E-09	6.195E-01
	C11orf21	1.310E-08	6.214E-01

	TUBB8P11	1.730E-08	6.195E-01
	DNPH1	1.770E-08	6.586E-01
Cluster4	*CTD-2017F17.2*	1.040E-06	6.210E-01
	GSE1	3.890E-06	6.249E-01
	GTPBP2	7.600E-06	6.518E-01
	RAB35	4.000E-05	6.477E-01
	RP11-331F4.4	5.500E-05	9.265E-01
	CELA3B	5.630E-05	8.204E-01
	LINC00294	7.630E-05	9.995E-01
	TMEM214	7.650E-05	8.894E-01
	AMY2A	8.880E-05	8.956E-01
	PAFAH1B2	1.097E-04	6.195E-01
	TPD52L2	1.123E-04	6.195E-01
	RP3-404F18.5	1.207E-04	7.419E-01
	RP11-346C20.3	1.327E-04	7.060E-01
	ANKDD1A	1.442E-04	6.195E-01
	RP11-229P13.2	1.584E-04	6.195E-01

	CTRB2	2.129E-04	9.906E-01
	CELA2A	2.147E-04	9.689E-01
	NT5C3AP1	2.364E-04	6.195E-01
	RMND5A	2.643E-04	7.008E-01
	SYCN	2.693E-04	9.345E-01
	C11orf53	2.890E-04	8.894E-01
	CTRB1	3.034E-04	8.768E-01
	BAK1P2	3.132E-04	6.247E-01
	NUP210P3	3.170E-04	6.195E-01
	EDEM2	3.345E-04	8.869E-01

Table S3 List of pathways discovered by the specific genes of each cluster by Reactome, which P-value is less than 0.05.

	**Pathway name**	**Entities P-value**	**Entities FDR**
Cluster1	LGI-ADAM interactions	3.940E-04	1.938E-01
	RUNX1 regulates transcription of genes involved in interleukin signaling	2.283E-03	4.264E-01
	NoRC negatively regulates rRNA expression	8.584E-03	4.264E-01
	Negative epigenetic regulation of rRNA expression	1.229E-02	4.264E-01

	Mitochondrial translation termination	1.473E-02	4.264E-01
	Mitochondrial translation elongation	1.473E-02	4.264E-01
	Mitochondrial translation initiation	1.578E-02	4.264E-01
	Mitochondrial translation	1.923E-02	4.264E-01
	Germ layer formation at gastrulation	2.405E-02	4.264E-01
	HDACs deacetylate histones	2.528E-02	4.264E-01
	COPI-independent Golgi-to-ER retrograde traffic	2.528E-02	4.264E-01
	Intestinal infectious diseases	2.929E-02	4.264E-01
	Prefoldin mediated transfer of substrate to CCT/TriC	3.402E-02	4.264E-01
	Estrogen-dependent nuclear events downstream of ESR-membrane signaling	3.402E-02	4.264E-01
	Regulation of RAS by GAPs	3.420E-02	4.264E-01
	Hedgehog 'off' state	3.565E-02	4.264E-01
	Defective ALG14 causes ALG14-CMS	3.886E-02	4.264E-01

	HSP90 chaperone cycle for steroid hormone receptors (SHR) in the presence of ligand	4.049E-02	4.264E-01
Cluster 2	Neutrophil degranulation	8.200E-04	3.346E-01
	tRNA processing in the mitochondrion	1.221E-03	3.346E-01
	Chemokine receptors bind chemokines	3.911E-03	5.558E-01
	rRNA processing in the mitochondrion	4.057E-03	5.558E-01
	VEGF ligand-receptor interactions	1.844E-02	7.448E-01
	VEGF binds to VEGFR leading to receptor dimerization	1.844E-02	7.448E-01
	Interleukin-4 and Interleukin-13 signaling	2.239E-02	7.448E-01
	HDL remodeling	2.475E-02	7.448E-01
	NR1H3 & NR1H2 regulate gene expression linked to cholesterol transport and efflux	2.915E-02	7.448E-01
	Signaling by VEGF	3.017E-02	7.448E-01
	Alpha-defensins	3.317E-02	7.448E-01
	Hypusine synthesis from eIF5A-lysine	3.882E-02	7.448E-01
	mRNA Editing	3.882E-02	7.448E-01

	Peptide ligand-binding receptors	3.951E-02	7.448E-01
	Cross-presentation of particulate exogenous antigens (phagosomes)	4.481E-02	7.448E-01
	Transcriptional regulation by the AP-2 (TFAP2) family of transcription factors	4.664E-02	7.448E-01
	DAP12 interactions	4.936E-02	7.448E-01
Cluster 3	RNA Polymerase I Promoter Opening	4.640E-04	2.766E-01
	DNA methylation	6.710E-04	2.766E-01
	Assembly of the ORC complex at the origin of replication	1.305E-03	2.766E-01
	Packaging Of Telomere Ends	2.193E-03	2.766E-01
	PRC2 methylates histones and DNA	2.344E-03	2.766E-01
	Condensation of Prophase Chromosomes	2.592E-03	2.766E-01
	SIRT1 negatively regulates rRNA expression	2.685E-03	2.766E-01
	Meiotic recombination	3.658E-03	2.766E-01
	Interleukin-1 processing	3.726E-03	2.766E-01
	ERCC6 (CSB) and EHMT2 (G9a)	3.942E-03	2.766E-01

	positively regulate rRNA expression		
	Transcriptional regulation of granulopoiesis	4.416E-03	2.766E-01
	Activated PKN1 stimulates transcription of AR (androgen receptor) regulated genes KLK2 and KLK3	4.449E-03	2.766E-01
	Recognition and association of DNA glycosylase with site containing an affected purine	4.610E-03	2.766E-01
	B-WICH complex positively regulates rRNA expression	5.580E-03	3.008E-01
	HDACs deacetylate histones	6.165E-03	3.008E-01
	Defective pyroptosis	6.273E-03	3.008E-01
	Recognition and association of DNA glycosylase with site containing an affected pyrimidine	6.801E-03	3.008E-01
	Nucleosome assembly	7.771E-03	3.008E-01
	Deposition of new CENPA-containing nucleosomes at the centromere	7.771E-03	3.008E-01
	RUNX1 regulates genes involved in megakaryocyte	8.266E-03	3.008E-01

	differentiation and platelet function		
	Defective CSF2RB causes SMDP5	8.596E-03	3.008E-01
	Defective CSF2RA causes SMDP4	8.596E-03	3.008E-01
	CLEC7A/inflammasome pathway	8.596E-03	3.008E-01
	Formation of the beta-catenin:TCF transactivating complex	8.986E-03	3.055E-01
	Cleavage of the damaged purine	9.684E-03	3.099E-01
	Depurination	1.082E-02	3.354E-01
	DNA Damage/Telomere Stress Induced Senescence	1.269E-02	3.807E-01
	Inhibition of DNA recombination at telomere	1.337E-02	3.877E-01
	RNA Polymerase I Promoter Escape	1.518E-02	4.250E-01
	Meiotic synapsis	1.655E-02	4.303E-01
	Cleavage of the damaged pyrimidine	1.797E-02	4.492E-01
	Depyrimidination	1.797E-02	4.492E-01
	Formation of annular gap junctions	1.996E-02	4.791E-01

	Senescence-Associated Secretory Phenotype (SASP)	2.166E-02	4.982E-01
	Meiosis	2.312E-02	5.085E-01
	Gap junction degradation	2.497E-02	5.243E-01
	Positive epigenetic regulation of rRNA expression	2.501E-02	5.253E-01
	HATs acetylate histones	3.004E-02	6.008E-01
	Amyloid fiber formation	4.409E-02	8.529E-01
	Base-Excision Repair, AP Site Formation	4.731E-02	8.529E-01
Cluster 4	Digestion	5.700E-05	2.171E-02
	Uptake of dietary cobalamins into enterocytes	7.930E-05	2.171E-02
	Digestion and absorption	1.070E-04	2.171E-02
	Digestion of dietary lipid	4.690E-04	7.125E-02
	Activation of Matrix Metalloproteinases	1.872E-03	2.265E-01
	Cobalamin (Cbl, vitamin B12) transport and metabolism	7.529E-03	4.694E-01
	Digestion of dietary carbohydrate	8.376E-03	4.694E-01

	Growth hormone receptor signaling	9.207E-03	4.694E-01
	Alpha-defensins	1.160E-02	4.694E-01
	CASP8 activity is inhibited	1.368E-02	4.694E-01
	Regulation of IFNG signaling	2.341E-02	4.694E-01
	Interleukin-6 signaling	2.618E-02	4.694E-01
	Prolactin receptor signaling	2.907E-02	4.694E-01
	CLEC7A (Dectin-1) induces NFAT activation	2.907E-02	4.694E-01
	Transcriptional regulation by RUNX3	3.076E-02	4.694E-01
	Caspase activation via Death Receptors in the presence of ligand	3.522E-02	4.694E-01
	ABC-family proteins mediated transport	3.680E-02	4.694E-01
	FOXO-mediated transcription of cell death genes	4.527E-02	4.694E-01

Table S4 List of pathways discovered by the specific genes of each cluster by KEGG, which P-value is less than 0.05.

	ID	Pathway name	P-value	P.adjust
Cluster 1	hsa05016	Huntington disease	4.343E-04	4.734E-02
Cluster 2	hsa05150	Staphylococcus aureus infection	8.209E-07	1.543E-04
	hsa04380	Osteoclast differentiation	6.862E-05	6.451E-03

	hsa04060	Cytokine-cytokine receptor interaction	3.207E-04	2.010E-02
	hsa04062	Chemokine signaling pathway	1.080E-03	4.065E-02
	hsa04145	Phagosome	1.256E-03	4.065E-02
	hsa05140	Leishmaniasis	1.429E-03	4.065E-02
	hsa04612	Antigen processing and presentation	1.514E-03	4.065E-02
	hsa04662	B cell receptor signaling pathway	1.891E-03	4.445E-02
	hsa04610	Complement and coagulation cascades	2.334E-03	4.875E-02
Cluster 3	hsa05418	Fluid shear stress and atherosclerosis	2.243E-06	4.958E-04
	hsa04145	Phagosome	3.376E-05	3.730E-03
	hsa04640	Hematopoietic cell lineage	3.454E-04	1.960E-02
	hsa04650	Natural killer cell mediated cytotoxicity	3.547E-04	1.960E-02
Cluster 4	hsa04972	Pancreatic secretion	5.675E-09	9.193E-07
	hsa04974	Protein digestion and absorption	1.525E-05	1.235E-03
	hsa04975	Fat digestion and absorption	3.499E-04	1.890E-02

Table S5 List of pathways discovered by the specific genes of each cluster by GO, which P-value is less than 0.05.

	ONTOLOGY	ID	Pathway name	P-value	P.adjust
Cluster 2	BP	GO:0043312	neutrophil degranulation	7.988E-10	1.289E-06
	BP	GO:0002283	neutrophil activation involved in immune response	8.921E-10	1.289E-06
	BP	GO:0002687	positive regulation of leukocyte migration	3.118E-06	2.528E-03
	BP	GO:0050921	positive regulation of chemotaxis	3.499E-06	2.528E-03
	BP	GO:1902105	regulation of leukocyte differentiation	6.212E-06	3.590E-03
	BP	GO:0002685	regulation of leukocyte migration	1.223E-05	5.688E-03
	BP	GO:0050727	regulation of inflammatory response	1.408E-05	5.688E-03
	BP	GO:0002690	positive regulation of leukocyte chemotaxis	1.615E-05	5.688E-03
	BP	GO:0097529	myeloid leukocyte migration	1.823E-05	5.688E-03
	BP	GO:0015939	pantothenate metabolic process	1.968E-05	5.688E-03
	BP	GO:0043254	regulation of protein-containing complex assembly	2.588E-05	6.799E-03
	BP	GO:0030041	actin filament polymerization	3.683E-05	8.869E-03

	BP	GO:0002526	acute inflammatory response	4.144E-05	9.212E-03
	BP	GO:1902107	positive regulation of leukocyte differentiation	5.896E-05	1.160E-02
	BP	GO:0090023	positive regulation of neutrophil chemotaxis	6.132E-05	1.160E-02
	BP	GO:0060326	cell chemotaxis	6.419E-05	1.160E-02
	BP	GO:0038112	interleukin-8-mediated signaling pathway	6.970E-05	1.185E-02
	BP	GO:0002688	regulation of leukocyte chemotaxis	8.389E-05	1.347E-02
	BP	GO:0098883	synapse pruning	9.052E-05	1.377E-02
	BP	GO:0071624	positive regulation of granulocyte chemotaxis	9.554E-05	1.380E-02
	BP	GO:0008154	actin polymerization or depolymerization	1.047E-04	1.441E-02
	BP	GO:0030833	regulation of actin filament polymerization	1.108E-04	1.456E-02
	BP	GO:0006766	vitamin metabolic process	1.184E-04	1.488E-02
	BP	GO:1902624	positive regulation of neutrophil migration	1.249E-04	1.504E-02
	BP	GO:0050920	regulation of chemotaxis	1.371E-04	1.585E-02
	BP	GO:0030595	leukocyte chemotaxis	1.512E-04	1.655E-02
	BP	GO:1902563	regulation of neutrophil activation	1.550E-04	1.655E-02
	BP	GO:0090022	regulation of neutrophil chemotaxis	1.604E-04	1.655E-02
	BP	GO:0035589	G protein-coupled purinergic nucleotide receptor signaling pathway	1.960E-04	1.689E-02
	BP	GO:0008064	regulation of actin polymerization or depolymerization	2.030E-04	1.689E-02
	BP	GO:0001810	regulation of type I hypersensitivity	2.079E-04	1.689E-02
	BP	GO:0016068	type I hypersensitivity	2.079E-04	1.689E-02
	BP	GO:0098758	response to interleukin-8	2.079E-04	1.689E-02
	BP	GO:0098759	cellular response to interleukin-8	2.079E-04	1.689E-02
	BP	GO:0150064	vertebrate eye-specific patterning	2.079E-04	1.689E-02
	BP	GO:0030832	regulation of actin filament length	2.104E-04	1.689E-02
	BP	GO:0030593	neutrophil chemotaxis	2.297E-04	1.794E-02

	BP	GO:2001028	positive regulation of endothelial cell chemotaxis	2.435E-04	1.852E-02
	BP	GO:1903706	regulation of hemopoiesis	2.948E-04	2.185E-02
	BP	GO:1903708	positive regulation of hemopoiesis	3.284E-04	2.359E-02
	BP	GO:0022407	regulation of cell-cell adhesion	3.347E-04	2.359E-02
	BP	GO:0043117	positive regulation of vascular permeability	3.595E-04	2.455E-02
	BP	GO:0006801	superoxide metabolic process	3.911E-04	2.455E-02
	BP	GO:0032535	regulation of cellular component size	3.924E-04	2.455E-02
	BP	GO:0043315	positive regulation of neutrophil degranulation	4.136E-04	2.455E-02
	BP	GO:0150062	complement-mediated synapse pruning	4.136E-04	2.455E-02
	BP	GO:1902565	positive regulation of neutrophil activation	4.136E-04	2.455E-02
	BP	GO:1902622	regulation of neutrophil migration	4.137E-04	2.455E-02
	BP	GO:0061844	antimicrobial humoral immune response mediated by antimicrobial peptide	4.162E-04	2.455E-02
	BP	GO:1903037	regulation of leukocyte cell-cell adhesion	4.654E-04	2.664E-02
	BP	GO:0019932	second-messenger-mediated signaling	4.702E-04	2.664E-02
	BP	GO:0150146	cell junction disassembly	5.061E-04	2.813E-02
	BP	GO:0043114	regulation of vascular permeability	5.401E-04	2.945E-02
	BP	GO:1990266	neutrophil migration	5.702E-04	3.052E-02
	BP	GO:0002761	regulation of myeloid leukocyte differentiation	5.954E-04	3.128E-02
	BP	GO:0001906	cell killing	6.232E-04	3.180E-02
	BP	GO:0002696	positive regulation of leukocyte activation	6.378E-04	3.180E-02
	BP	GO:0030225	macrophage differentiation	6.382E-04	3.180E-02
	BP	GO:0038033	positive regulation of endothelial cell chemotaxis by VEGF-activated vascular endothelial growth factor receptor signaling	6.855E-04	3.292E-02

			pathway		
	BP	GO:1905907	negative regulation of amyloid fibril formation	6.855E-04	3.292E-02
	BP	GO:0071621	granulocyte chemotaxis	7.047E-04	3.292E-02
	BP	GO:0006767	water-soluble vitamin metabolic process	7.397E-04	3.292E-02
	BP	GO:0032271	regulation of protein polymerization	7.467E-04	3.292E-02
	BP	GO:0002673	regulation of acute inflammatory response	7.484E-04	3.292E-02
	BP	GO:0002429	immune response-activating cell surface receptor signaling pathway	7.518E-04	3.292E-02
	BP	GO:0002757	immune response-activating signal transduction	7.518E-04	3.292E-02
	BP	GO:1903039	positive regulation of leukocyte cell-cell adhesion	8.347E-04	3.600E-02
	BP	GO:0050867	positive regulation of cell activation	8.584E-04	3.627E-02
	BP	GO:0014068	positive regulation of phosphatidylinositol 3-kinase signaling	8.659E-04	3.627E-02
	BP	GO:0071622	regulation of granulocyte chemotaxis	9.378E-04	3.872E-02
	BP	GO:0051258	protein polymerization	9.797E-04	3.938E-02
	BP	GO:0045785	positive regulation of cell adhesion	9.812E-04	3.938E-02
	BP	GO:0007159	leukocyte cell-cell adhesion	1.011E-03	4.003E-02
	BP	GO:0002438	acute inflammatory response to antigenic stimulus	1.025E-03	4.003E-02
	BP	GO:0050808	synapse organization	1.078E-03	4.152E-02
	BP	GO:0071216	cellular response to biotic stimulus	1.120E-03	4.260E-02
	BP	GO:2001026	regulation of endothelial cell chemotaxis	1.158E-03	4.345E-02
	BP	GO:1902903	regulation of supramolecular fiber organization	1.215E-03	4.500E-02
	BP	GO:0031333	negative regulation of protein-containing complex assembly	1.259E-03	4.605E-02

	BP	GO:0050728	negative regulation of inflammatory response	1.337E-03	4.830E-02
	BP	GO:0048010	vascular endothelial growth factor receptor signaling pathway	1.404E-03	4.899E-02
	BP	GO:0002886	regulation of myeloid leukocyte mediated immunity	1.414E-03	4.899E-02
	BP	GO:1903793	positive regulation of anion transport	1.414E-03	4.899E-02
	BP	GO:0038089	positive regulation of cell migration by vascular endothelial growth factor signaling pathway	1.424E-03	4.899E-02
	BP	GO:0006909	phagocytosis	1.451E-03	4.932E-02
	CC	GO:0070820	tertiary granule	1.787E-07	5.147E-05
	CC	GO:0030667	secretory granule membrane	4.233E-07	6.096E-05
	CC	GO:0070821	tertiary granule membrane	3.884E-05	3.729E-03
	MF	GO:0017159	pantetheine hydrolase activity	6.382E-07	2.929E-04
	MF	GO:0140375	immune receptor activity	2.701E-06	6.200E-04
	MF	GO:0019865	immunoglobulin binding	5.036E-05	7.705E-03
	MF	GO:0004918	interleukin-8 receptor activity	7.460E-05	8.560E-03
	MF	GO:0019864	IgG binding	1.001E-04	9.186E-03
	MF	GO:0045028	G protein-coupled purinergic nucleotide receptor activity	2.166E-04	1.459E-02
	MF	GO:0019959	interleukin-8 binding	2.225E-04	1.459E-02
	MF	GO:0033218	amide binding	4.983E-04	2.795E-02
	MF	GO:0001540	amyloid-beta binding	5.480E-04	2.795E-02
	MF	GO:0019763	immunoglobulin receptor activity	7.333E-04	2.895E-02
	MF	GO:0001614	purinergic nucleotide receptor activity	7.568E-04	2.895E-02
	MF	GO:0016502	nucleotide receptor activity	7.568E-04	2.895E-02
	MF	GO:0019958	C-X-C chemokine binding	1.094E-03	3.861E-02
	MF	GO:0004896	cytokine receptor activity	1.556E-03	4.903E-02
	MF	GO:0044183	protein folding chaperone	1.602E-03	4.903E-02
Cluster 3	BP	GO:0002699	positive regulation of immune effector process	6.470E-07	2.038E-03
	BP	GO:0006909	phagocytosis	1.126E-06	2.038E-03

	BP	GO:0042110	T cell activation	7.892E-06	8.211E-03
	BP	GO:0031343	positive regulation of cell killing	9.108E-06	8.211E-03
	BP	GO:0002703	regulation of leukocyte mediated immunity	1.134E-05	8.211E-03
	BP	GO:0031341	regulation of cell killing	1.668E-05	1.006E-02
	BP	GO:0002221	pattern recognition receptor signaling pathway	3.758E-05	1.622E-02
	BP	GO:1903557	positive regulation of tumor necrosis factor superfamily cytokine production	3.919E-05	1.622E-02
	BP	GO:0002705	positive regulation of leukocyte mediated immunity	4.994E-05	1.622E-02
	BP	GO:0001906	cell killing	5.104E-05	1.622E-02
	BP	GO:0001909	leukocyte mediated cytotoxicity	5.338E-05	1.622E-02
	BP	GO:0010922	positive regulation of phosphatase activity	5.377E-05	1.622E-02
	BP	GO:0050863	regulation of T cell activation	6.068E-05	1.690E-02
	BP	GO:0043312	neutrophil degranulation	1.052E-04	2.720E-02
	BP	GO:0002283	neutrophil activation involved in immune response	1.133E-04	2.733E-02
	BP	GO:0034125	negative regulation of MyD88-dependent toll-like receptor signaling pathway	1.480E-04	2.820E-02
	BP	GO:0034758	positive regulation of iron ion transport	1.480E-04	2.820E-02
	BP	GO:0034761	positive regulation of iron ion transmembrane transport	1.480E-04	2.820E-02
	BP	GO:1904440	positive regulation of iron ion import across plasma membrane	1.480E-04	2.820E-02
	BP	GO:0050766	positive regulation of phagocytosis	1.792E-04	3.191E-02
	BP	GO:0050764	regulation of phagocytosis	1.851E-04	3.191E-02
	BP	GO:0002697	regulation of immune effector process	2.231E-04	3.671E-02
	BP	GO:1902105	regulation of leukocyte	2.425E-04	3.816E-02

			differentiation		
	BP	GO:0030217	T cell differentiation	2.883E-04	4.139E-02
	BP	GO:0001910	regulation of leukocyte mediated cytotoxicity	3.071E-04	4.139E-02
	BP	GO:0050870	positive regulation of T cell activation	3.071E-04	4.139E-02
	BP	GO:0002448	mast cell mediated immunity	3.176E-04	4.139E-02
	BP	GO:0002708	positive regulation of lymphocyte mediated immunity	3.201E-04	4.139E-02
	BP	GO:0032760	positive regulation of tumor necrosis factor production	3.494E-04	4.362E-02
	BP	GO:0001912	positive regulation of leukocyte mediated cytotoxicity	4.203E-04	4.689E-02
	BP	GO:0046631	alpha-beta T cell activation	4.304E-04	4.689E-02
	BP	GO:0060557	positive regulation of vitamin D biosynthetic process	4.404E-04	4.689E-02
	BP	GO:0060559	positive regulation of calcidiol 1-monooxygenase activity	4.404E-04	4.689E-02
	BP	GO:1904438	regulation of iron ion import across plasma membrane	4.404E-04	4.689E-02
	BP	GO:1903555	regulation of tumor necrosis factor superfamily cytokine production	4.722E-04	4.884E-02
	CC	GO:0042101	T cell receptor complex	1.825E-06	5.653E-04
	CC	GO:0030139	endocytic vesicle	2.812E-06	5.653E-04
	CC	GO:0045334	clathrin-coated endocytic vesicle	1.027E-04	1.376E-02
	CC	GO:0098802	plasma membrane signaling receptor complex	1.651E-04	1.659E-02
	MF	GO:0030898	actin-dependent ATPase activity	8.167E-06	4.574E-03
	MF	GO:0000146	microfilament motor activity	2.174E-05	6.086E-03
Cluster 4	MF	GO:0004252	serine-type endopeptidase activity	1.799E-06	6.015E-04
	MF	GO:0008236	serine-type peptidase activity	4.137E-06	6.015E-04

	MF	GO:0017171	serine hydrolase activity	4.917E-06	6.015E-04
	MF	GO:0004175	endopeptidase activity	6.790E-06	6.230E-04
	BP	GO:0007586	digestion	3.985E-06	1.011E-02
	BP	GO:0009235	cobalamin metabolic process	1.149E-05	1.458E-02
	BP	GO:0002474	antigen processing and presentation of peptide antigen via MHC class I	4.642E-05	3.060E-02
	BP	GO:0033013	tetrapyrrole metabolic process	4.823E-05	3.060E-02

Table S6 List of pathways discovered by the top 25 genes of each cluster by Reactome, which P-value is less than 0.05.

	**Pathway name**	**Entities pValue**	**Entities FDR**
Cluster 1	Transcriptional regulation of pluripotent stem cells	3.070E-03	2.390E-01
	POU5F1 (OCT4), SOX2, NANOG repress genes related to differentiation	1.810E-02	2.390E-01
	Metallothioneins bind metals	2.870E-02	2.390E-01
	POU5F1 (OCT4), SOX2, NANOG activate genes related to proliferation	3.760E-02	2.390E-01
	Response to metal ions	3.760E-02	2.390E-01

	Microtubule-dependent trafficking of connexons from Golgi to the plasma membrane	3.930E-02	2.390E-01
	Transport of connexons to the plasma membrane	4.110E-02	2.390E-01
	Post-chaperonin tubulin folding pathway	4.460E-02	2.390E-01
Cluster 2	Transcriptional regulation by the AP-2 (TFAP2) family of transcription factors	5.130E-06	5.640E-04
	VEGF ligand-receptor interactions	1.450E-04	5.200E-03
	VEGF binds to VEGFR leading to receptor dimerization	1.450E-04	5.200E-03
	Signaling by VEGF	2.230E-04	6.030E-03
	Regulation of gene expression by Hypoxia-inducible Factor	5.030E-04	1.110E-02
	TFAP2 (AP-2) family regulates transcription of growth factors and their receptors	9.790E-04	1.640E-02

	Interleukin-4 and Interleukin-13 signaling	1.090E-03	1.640E-02
	Signaling by Receptor Tyrosine Kinases	1.900E-03	2.480E-02
	Glucagon-type ligand receptors	2.670E-03	2.930E-02
	Vitamin B5 (pantothenate) metabolism	2.670E-03	2.930E-02
	nucleus signaling by ERBB4	4.730E-03	4.730E-02
	NR1H3 & NR1H2 regulate gene expression linked to cholesterol transport and efflux	9.100E-03	8.190E-02
	Signaling by ERBB4	1.370E-02	1.030E-01
	NR1H2 and NR1H3-mediated signaling	1.470E-02	1.030E-01
	Cellular response to hypoxia	1.500E-02	1.050E-01
	Chylomicron clearance	1.940E-02	1.160E-01
	Class B/2 (Secretin family receptors)	1.960E-02	1.180E-01
	Insulin-like Growth Factor-2 mRNA Binding Proteins (IGF2BPs/IMPs/VICKZs) bind RNA	2.790E-02	1.390E-01

	Cross-presentation of particulate exogenous antigens (phagosomes)	2.790E-02	1.390E-01
	Chylomicron assembly	3.000E-02	1.500E-01
	VEGFA-VEGFR2 Pathway	3.100E-02	1.500E-01
	Chylomicron remodeling	3.630E-02	1.500E-01
	Platelet degranulation	3.760E-02	1.500E-01
	Response to elevated platelet cytosolic Ca2+	4.100E-02	1.530E-01
	ADORA2B mediated anti-inflammatory cytokines production	4.660E-02	1.530E-01
	Metabolism of vitamins and cofactors	4.850E-02	1.530E-01
	Signaling by Interleukins	4.920E-02	1.530E-01
Cluster 3	ATF4 activates genes in response to endoplasmic reticulum stress	1.780E-03	1.560E-01
	PERK regulates gene expression	2.690E-03	1.560E-01

	FOXO-mediated transcription of oxidative stress, metabolic and neuronal genes	3.630E-03	1.560E-01
	FOXO-mediated transcription	1.710E-02	1.810E-01
Cluster 4	Digestion of dietary carbohydrate	3.640E-04	1.490E-02
	Cobalamin (Cbl, vitamin B12) transport and metabolism	1.100E-03	1.860E-02
	Digestion	1.430E-03	1.860E-02
	Digestion and absorption	2.080E-03	2.080E-02
	Activation of Matrix Metalloproteinases	2.670E-03	2.140E-02
	Metabolism of water-soluble vitamins and cofactors	3.420E-02	2050E-01
	Degradation of the extracellular matrix	4.140E-02	2.070E-01
	ER Quality Control Compartment (ERQC)	4.750E-02	2.170E-01

For instance, the most significant pathway (p-value = 5.13E-6) related to Cluster 2 is “Transcription regulation by the AP-2 (TFAP2) family of transcription factors”. Five proteins (TFAP2A, TFAP2B, TFAP2C, TFAP2D, TFAP2E) are included in the AP-2 family of transcription factors. The AP-2

family of transcription factors regulates the gene expression by interacting with the transcriptional co-factors of the CITED family and recruiting transcription co-activators to TFAP2-bound DNA elements. The AP-2 family relates to breast cancer and mammary development. De et al. [1] had reported that TFAP2C regulates EGFR in luminal breast cancer as well as HER2 breast cancer. Cyr et al. [2] had reported that the knockdown of TFAP2C in luminal breast carcinoma cells induces the loss of luminal-associated gene expression in contrast to the gain of the basal-associated gene expression. It also pointed out that TFAP2C is the key factor to maintaining the luminal breast carcinoma, and Tcfap2c influences the luminal cells in the mammary. While Bogachek et al. [3] had reported that the sumoylation of TFAP2A maintains the basal breast cancer phenotype. Also, the most significant pathway (p-value = 1.78E-3) related to Cluster 3 is "ATF4 activates genes in response to endoplasmic reticulum stress". ATF4 is a transcription factor and ATF4 activates 6 genes expression including IL-8, MCP1, IGFBP-1, CHOP, HERP1, and ATF3. Marchand et al. [4] had reported that insulin-like growth factor binding protein 1 (IGFBP1) is induced during the Endoplasmic reticulum (ER) stress in human hepatocytes. It pointed out that the region of the human IGFBP1 gene containing an activating transcription factor 4 (ATF4) composite site is required for the activation of ER stress. Armstrong et al. [5] had reported that ATF4 induces the ER stress-induced cell death of neuroectodermal tumor cells which induced by the chemotherapeutic agents fenretinide and bortezomib.

1. De-Andrade, J.P.; Park, J.M.; Gu, V.W.; et al. EGFR Is Regulated by TFAP2C in Luminal Breast Cancer and Is a Target for VandetanibTFAP2C Regulates Breast Cancer through EGFR. Molecular cancer therapeutics 2016, 15(3): 503-511. https://doi.org/10.1158/1535-7163.MCT-15-0548-T

2. Cyr, A.R.; Kulak, M.V.; Park, J.M.; et al. TFAP2C governs the luminal epithelial phenotype in mammary development and carcinogenesis. Oncogene 2015, 34(4): 436-444. https://doi.org/10.1038/onc.2013.569

3. Bogachek, M.V.; Chen, Y.; Kulak, M.V.; et al. Sumoylation pathway is required to maintain the basal breast cancer subtype. Cancer cell 2014, 25(6): 748-761. https://doi.org/10.1016/j.ccr.2014.04.008

4. Marchand, A.; Tomkiewicz, C.; Magne, L.; et al. Endoplasmic reticulum stress induction of insulin-like growth fac-tor-binding protein-1 involves ATF4. Journal of Biological Chemistry 2006, 281(28): 19124-19133. https://doi.org/10.1074/jbc.M602157200

5. Armstrong, J.L.; Flockhart, R.; Veal, G.J.; et al. Regulation of endoplasmic reticulum stress-induced cell death by ATF4 in neuroectodermal tumor cells. Journal of Biological Chemistry 2010, 285(9): 6091-6100. https://doi.org/10.1074/jbc.M109.014092
